# Integration of Small RNA and Degradome Sequencing Reveals the Regulatory Network of Al-Induced Programmed Cell Death in Peanut

**DOI:** 10.3390/ijms23010246

**Published:** 2021-12-27

**Authors:** Bin Tong, Yusun Shi, Aaron Ntambiyukuri, Xia Li, Jie Zhan, Aiqin Wang, Dong Xiao, Longfei He

**Affiliations:** 1 National Demonstration Center for Experimental Plant Science Education, College of Agriculture, Guangxi University, Nanning 530004, China; 1917301034@st.gxu.edu.cn (B.T.); shiyusun2012@gmail.com (Y.S.); aaronyaron2013@gmail.com (A.N.); 2017401005@st.gxu.edu.cn (X.L.); may2399@163.com (J.Z.); waiqing1966@126.com (A.W.); 2 Guangxi Key Laboratory for Agro-Environment and Agro-Product Safety, Nanning 530004, China; 3 Key Laboratory of Crop Cultivation and Tillage, Guangxi University, Nanning 530004, China

**Keywords:** peanut, microRNAs, degradome, aluminum stress, programmed cell death, regulatory network

## Abstract

Peanut is one of the most important oil crops in the world. In China, the peanut is highly produced in its southern part, in which the arable land is dominated by acid soil. At present, miRNAs have been identified in stress response, but their roles and mechanisms are not clear, and no miRNA studies have been found related to aluminum (Al)-induced programmed cell death (PCD). In the present study, transcriptomics, sRNAs, and degradome analysis in the root tips of two peanut cultivars ZH2 (Al-sensitive, S) and 99-1507 (Al-tolerant, T) were carried out. Here, we generated a comprehensive resource focused on identifying key regulatory miRNA-target circuits that regulate PCD under Al stress. Through deep sequencing, 2284 miRNAs were identified and 147 miRNAs were differentially expressed under Al stress. Furthermore, 19237 target genes of 749 miRNAs were validated by degradome sequencing. GO and KEGG analyses of differential miRNA targets showed that the pathways of synthesis and degradation of ketone bodies, citrate cycle (TCA cycle), and peroxisome were responded to Al stress. The combined analysis of the degradome data sets revealed 89 miRNA-mRNA interactions that may regulate PCD under Al stress. Ubiquitination may be involved in Al-induced PCD in peanut. The regulatory networks were constructed based on the differentially expressed miRNAs and their targets related to PCD. Our results will provide a useful platform to research on PCD induced by Al and new insights into the genetic engineering for plant stress response.

## 1. Introduction

Aluminum (Al) toxicity is a worldwide agronomic problem that limits crop production in acidic soils. The response of plant roots to Al is mainly caused by Al exclusion mechanisms and Al tolerance mechanisms [1]. The mechanism of Al exclusion mainly includes the secretion of organic acids (citrate, malate, and oxalate) [2], the increase of rhizosphere pH [3], and the binding of the cell wall to Al [4]. The mechanisms of Al tolerance mainly include the regionalization of Al, the inactivation of toxicity after the chelation of Al with organic acids in cells, in order to reduce the toxicity of Al to plants and improve plant resistance [5]. It was found that Al stress could induce the changes in antioxidant enzyme activities in plants, including superoxide dismutase (SOD), peroxidase (POD), catalase (CAT), ascorbate peroxidase (APX), etc. [6]. 

Al can induce plant cells to produce reactive oxygen species (ROS) and activate some antioxidant enzymes. A high concentration of ROS resulted in membrane lipid peroxidation and increased membrane permeability, resulting in cell death [7]. The signal pathway of PCD induced by Al is still unclear. ROS, NO, and Ca^2 +^ may be the signal molecules of PCD [8]. In recent years, some progress has been made in the study of signal molecules, proteins, and genes related to Al-induced PCD in plants, but the exact mechanisms, such as the relationship between the difference of PCD in different plants and Al tolerance in plants, are still unclear. It is not known whether there are universal regulatory pathways or mechanisms for Al-induced PCD related signaling factors, etc. 

The microRNA (miRNA) is a kind of endogenous non-coding RNA with regulatory function and its size is about 20–25 nucleotides. Target genes expression can be regulated by transcription and post-transcriptional regulation. It plays an important role in plant growth, development, and stress response. The target genes mainly include transcription factors, signal proteins, enzymes, etc. The recently available continuous updates of sequencing platforms have made possible the large-scale association analysis of mRNA and small RNA (sRNA) in non-model plants [9]. Currently, many potential heavy metal-responsive miRNA candidates have been identified in rice, soybean, Arabidopsis, and many other plants [10,11,12,13]. In the last few years, some potential Al stress-responsive miRNAs have been discovered through whole-genome sequencing. A group of new miRNAs was identified in *Medicago truncatula* by a deep-sequencing method and a number of miRNAs induced by Al stress was discovered [14]. Details on how the miRNAs regulate downstream gene expression involved in Al tolerance remain unclear. 

In flowering plants, miR159 can inhibit GA regulated PCD in tapetum cells, by inhibiting GAMYBs transcription factor [15]. The miR164 negatively regulates NAC transcription factor ORE1, while ORE1 positively regulates age-induced PCD in Arabidopsis leaves [15]. The regulatory mechanism of miR398 included the degradation and translation inhibition of Cu/Zn superoxide dismutase mRNA [16], and superoxide dismutase may be involved in Al-induced PCD. Some miRNAs, such as miR319, may directly affect the expression of metacaspase. The decrease of miR319a.2 expression under Al stress may lead to the increase of PCD [16]. The high-throughput analysis of these endogenous conserved miRNAs under heavy metals stress may provide new insights into the mechanism of the plant stress response [17]. Details on how the miRNAs regulate downstream gene expression involved in Al tolerance require further investigation in future studies. 

Plant miRNAs complement target genes completely or nearly completely, and cut target genes to inhibit gene expression. Using bioinformatics methods to predict target genes cannot determine whether the predicted target genes are true. In addition, if there is a high mismatch between miRNA and target genes, bioinformatics prediction will lose many real target genes. Degradome sequencing integrates high-throughput sequencing, RLM-5 ‘race, and bioinformatics analysis to identify miRNA target genes in the whole genome. Through comparison and analysis of sequencing data, we can find that a peak will appear at a site of mRNA sequence, which is a candidate miRNA splicing site. Degradome sequencing enabled researchers to dispose of the limitations of bioinformatics prediction of false positive, and really found the target gene of miRNA splicing from the experiment. Therefore, a large number of miRNA target genes have been identified in many plants by degradome sequencing technology, such as rice [18], wheat [19], sesame [20], and cotton [21], etc.

Peanut is an important oil and energy crop in the world. Al inhibits the growth and development of peanut and this inhibition results in reduced peanut production [22,23]. A more detailed comparative transcriptome analysis for the duration of Al stress is still needed to further understand the mechanisms of Al toxicity and tolerance. The knowledge regarding the miRNA expression profile in peanut under heavy metals stress is still limited. Therefore, it is necessary to comprehensively identify miRNAs related to heavy metals absorption and tolerance in peanut.

Our purpose in this study is to systematically identify potential Al-responsive miRNAs and their target genes in peanut. Here, the transcriptome data that were previously conducted by Xiao et al. [24] were used as a reference sequence for miRNA and degradome sequencing analyses in peanut. The combination of sRNA, degradome, and transcriptome has produced a comprehensive resource, focusing on identifying the key regulatory pathways of miRNAs that could induce PCD under Al stress and provide useful information for the improvement of peanut plants. 

## 2. Results

### 2.1. Sequencing and Identification of Known and Novel miRNAs

**Figure 1:**
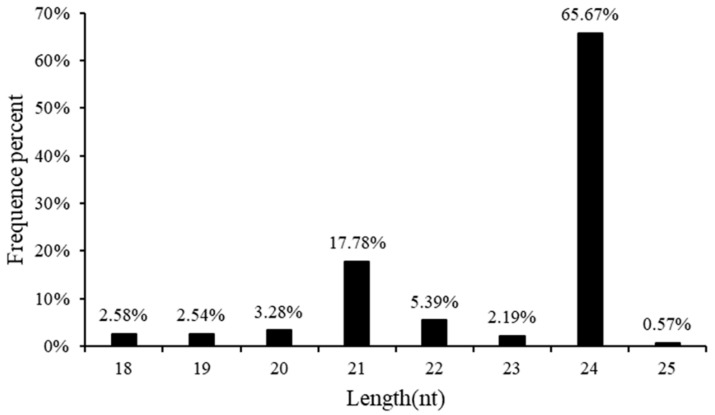
Length distribution and abundance of the sequences.

ZH2 (Al-sensitive, S) and 99-1507(Al-tolerant, T) genotypes were used to generate 26 small RNA libraries representing three controls without Al stress at different time points (0, 8, and 24 h-0) and two Al treatments at different time points (8 and 24 h) for high-throughput sequencing (Table S1). The bioinformatics analysis of our data was performed using the development pipeline (Figure S1). After removing low-quality sequences, 18 to 25 nt long sequences were obtained. The size distributions of the total and unique sRNAs were summarized in Figure S2. There was no significant difference among the 26 libraries. The displayed length of these peanut sRNAs ranged from 18 to 25 nt, and the sRNA with 24 nt showed the highest redundancy size category among all of the 26 libraries (Figure S2 and Figure 1). It revealed that 65.67% of all the miRNAs were 24 nt in length (Figure 1).

**Figure 2:**
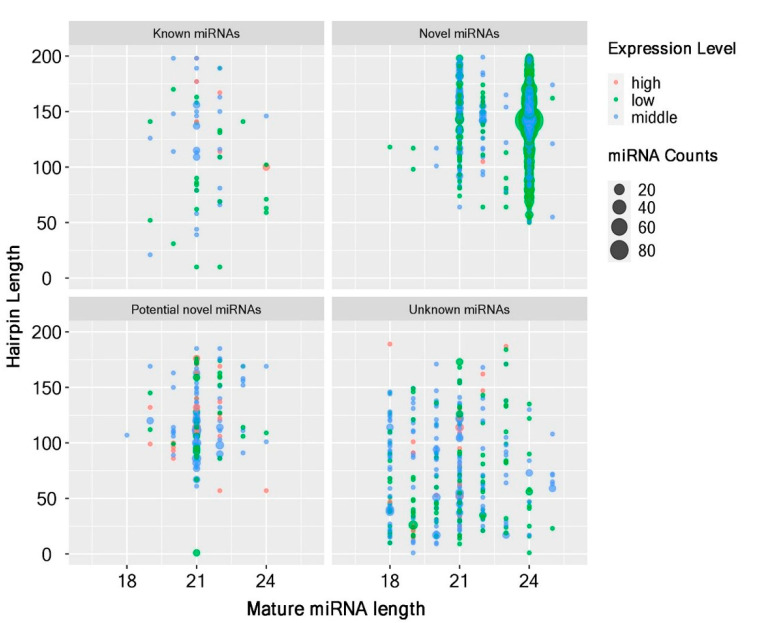
The four indicated groups of miRNAs identified from an incompatible interaction. The miRNA counts, the counts of miRNAs from miRBase. Expression level, low indicates <10, middle indicates >10, but less than average, high indicates over average. Hairpin length, pre-miRNA length.

We used the BLAST algorithm to query all of the clean and unique sRNA read sequences and searched for related non-coding RNAs stored in Rfam and NCBI GenBank databases then removed rRNA, tRNA, snRNA, and snoRNA sequences. In order to predict new miRNAs, the remaining unannotated sRNA unique reads that do not match with any transcriptome library were searched, and the secondary structure prediction was performed. All of the sRNAs loci that could be folded into secondary structure were considered as potential new miRNA candidates. A total of 2284 miRNAs were identified, including 1670 novel (gp4), 83 known (gp1a), 185 potential novel miRNAs (predicted but not mapped in the plant genome, gp1b, 2a), and 346 unknown miRNAs (mapped on the plant genome but not in the miRNA database, gp2b, 3; Figure 2 and Table S2). These miRNAs belong to 49 recorded miRNA families (Figure S3). 

### 2.2. Al-Responsive miRNAs in Peanut 

**Figure 3:**
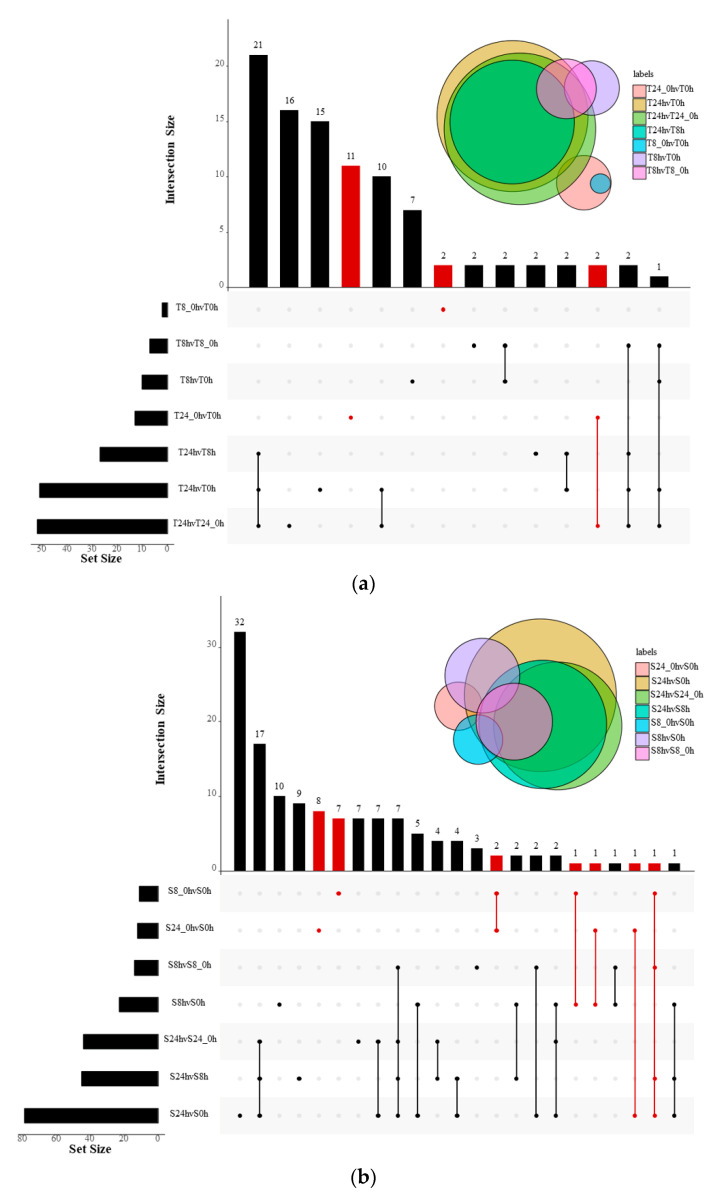
Al-responsive miRNAs in peanut. Upset of differentially expressed genes in response to 99-1507 (**a**) and ZH2 (**b**). UpSetR visualizes intersections of sets as a matrix, in which the rows represent the sets and the columns represent their intersections. Refer to Conway, J.R. et al. [25] for legend description. The element query (red) selects classified as deletions.

**Figure 4:**
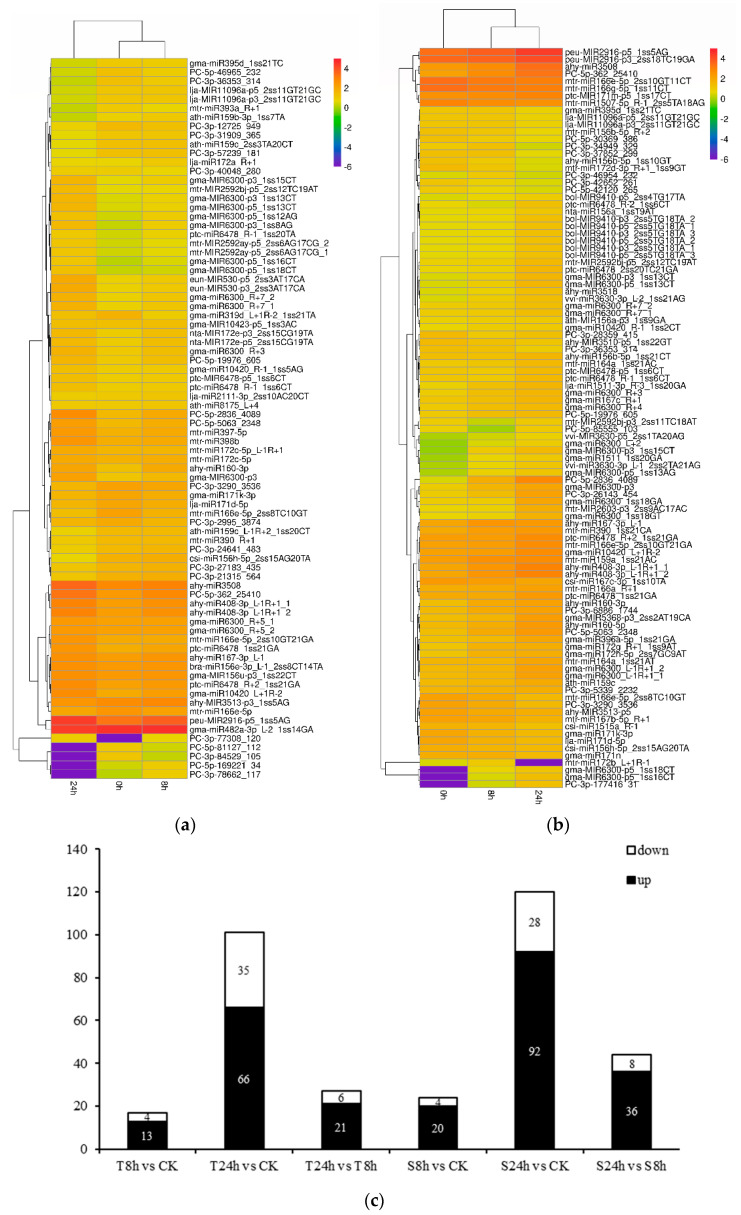
The numbers and expression profiling of differentially expressed miRNAs in root tips of peanut. Hierarchical clustering of differentially expressed miRNAs in 99-1507 (**a**) and ZH2 (**b**) at three different Al treatment periods (0, 8, and 24 h). Red indicates upregulated miRNAs, while purple indicates downregulated miRNAs. The original expression values of the miRNAs were normalized using Z-score normalization. The absolute signal intensity ranges from −6.0 to +4.0, with corresponding color changes from purple to red. (**c**) The number of differentially expressed miRNAs under Al stress compared with the control.

To identify miRNAs responsive to Al stress in peanut, the differential expression of miRNAs in the 26 libraries was analyzed and compared using the read counts generated from the high-throughput sequencing. In the experiment, the controls (0, 8, and 24 h-0) were set up. After removing the influence of unrelated factors, 147 miRNAs (|log2 fold change| ≥ 1 and *p* < 0.05) showed differential expression patterns, where 45 were only differentially expressed in 99-1507 (T), 67 only in ZH2(S), and 35 in both cultivars (Figure 3, Table S3). These miRNAs belong to 38 families. The miR6300 was the largest miRNA family with 20 members responsive to Al stress, followed by miR156 and miR172 families (Table S3). The heatmaps of differentially expressed miRNAs were illustrated in Figure 4a,b. The gma-MIR6300-p3_1ss15CT, csi-miR156h-5p_2ss15AG20TA, ath-miR159b-3p_1ss7TA, ath-miR159c_2ss3TA20CT, PC-5p-2836_4089 and PC-3p-36353_314 were the most responsive to Al stress. Among them, gma-MIR6300-p3_1ss15CT and PC-5p-2836_4089 were upregulated significantly, while csi-miR156h-5p_2ss15AG20TA, ath-miR159b-3p_1ss7TA, ath-miR159c_2ss3TA20CT, and PC-3p-36353_314 were downregulated significantly (Figure 4a,b, Table S3). 

In addition to the validation of differentially expressed miRNAs, we also examined the distribution of 147 differential miRNAs between the control and treatments (Figure 4c). Compared with the control, the largest number (92 miRNAs) was upregulated miRNAs in ZH2 after 24 h of Al treatment, while 35 miRNAs were significantly downregulated in 99-1507 after 24 h of Al treatment (Figure 4c). It was found that the same miRNA could show different expression patterns up or downregulation in different comparisons. For example, PC-3p-46954_232 was upregulated at 8 h in ZH2 but downregulated at 24 h in ZH2, PC-5p-85555_103 was downregulated at 8 h in ZH2 but was upregulated at 24 h in ZH2 (Figure 4b). These results indicated that the expression levels of miRNAs may depend on the duration of Al treatment. 

### 2.3. Target Prediction of the Known and Novel miRNAs by Degradome Sequencing

To research on the functions of miRNA, we first determined the targets of miRNA. Through degradome sequencing, 7.97 million raw reads representing 3.39 million unique reads, were generated from the six mixed degradome pools (Table S4). After removing the reads lacking the adaptor and the low-quality sequences, 5.66 million (71.0% of all reads) sequences were successfully mapped to peanut, containing 2.16 million unique reads (Table S4). 

Through degradome analysis, a total of 19237 targets were identified for 749 miRNAs (Table S5). Generally, many miRNAs targeted multiple genes and most of them were highly conserved. Different members of miRNA families cleaved the same target genes or different members of the same gene family. For instance, 68 targets were identified to be cleaved by gma-miR172g_R+1_1ss9AT, and 67 targets were identified to be cleaved by mtr-miR1507-5p_R-1_2ss5TA18AG, while eight miRNAs were detected to cleave only one transcript target (XM_016096779.2), which was annotated to a protein related to the heat shock. In addition, degradome analysis showed that 8192 genes were the targets of 147 differentially expressed miRNAs in peanut (Table S6). Through the expression analysis of target genes in response to Al stress in peanut, a large number of members of the WRKY, ABC transporters, NAC, MYB, and TCP families were ranked in the context of Al response, indicating their important roles for peanut in combating Al stress (Table S6). Generally, many miRNAs targeted PCD-related genes and stress-related genes. For instance, metacaspases (MCs) observed in the study were targeted by both gma-miR396a-5p_1ss21GA and mtr-MIR2592bj-p3_2ss11TC18AT, and miR10420 members targeted SODs, PODs, RBOHs, and APXs, etc. (Table S7). 

### 2.4. Annotation and Enrichment Analysis of Targets for miRNAs under Al Stress

**Figure 5:**
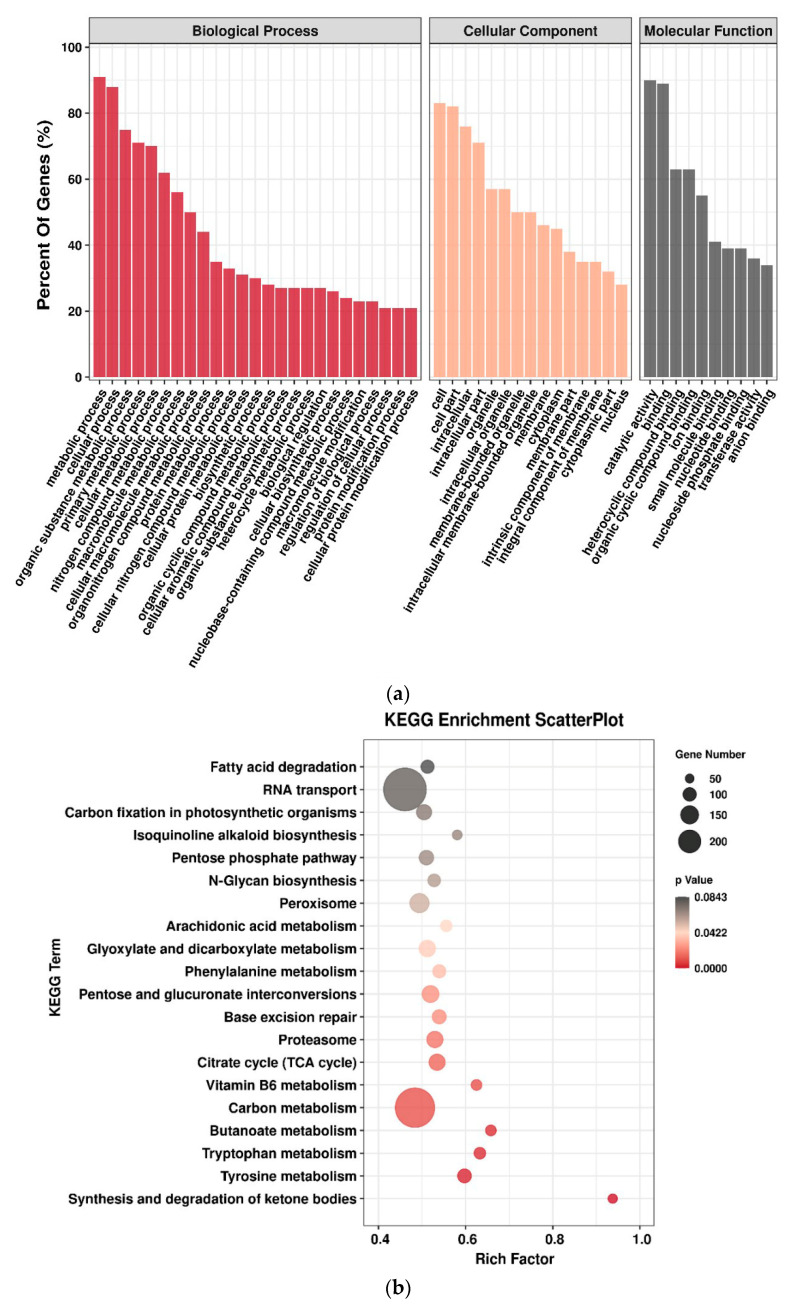
Gene ontology classification (**a**) and KEGG pathway enrichment (**b**) of target genes for different miRNAs.

To research the regulatory functions of miRNAs in response to Al stress in peanut, gene ontology (GO) functional classification and Kyoto encyclopedia of genes and genomes (KEGG) pathway enrichment were conducted to further understand the functions (Figure 5). Out of the 749 genes, differential miRNAs targeted 8215 identified target genes, and the GO terms were assigned to 6724 genes. For these 6724 genes, a total of 4387 GO terms were obtained. It was seen that the GO terms for differential miRNAs were uniformly assigned to each of the biological processes (2540), molecular function (553), and cellular component (1294) categories.

First, GO functional classification showed that the most enriched biological processes were with the two most frequent categories as the metabolic process and biosynthetic process, such as the organic substance metabolic process (GO:0071704), nitrogen compound metabolic process (GO:0006807), macromolecule metabolic process (GO:0043170), organic substance biosynthetic process (GO:1901576), and cellular biosynthetic process (GO:0044249) (Figure 5a). Among the cellular components, the most significant GO terms were involved in cell (GO:0005623), organelle (GO:0043226), membrane (GO:0016020), and cytoplasm (GO:0005737) (Figure 5a). For molecular functions, genes were mainly involved in binding subclasses, such as small molecule binding (GO:0036094), nucleotide binding (GO:0000166), ribonucleoside binding (GO:0032549), and GTP binding (GO:0005525). Another important part of the molecular function was enzyme activity, for instance, carbonyl reductase (NADPH) activity (GO:0004090), oxidoreductase activity (GO:0016639), and endopeptidase activity (GO:0004175). 

Second, based on the KEGG analysis, the most enriched pathways in response to Al stress were those related to the synthesis and degradation of ketone bodies (ko00072), tyrosine metabolism (ko00350), tryptophan metabolism (ko00380), butanoate metabolism (ko00650), and carbon metabolism (ko00710) (Figure 5b). Previous studies have inferred that miR159, miR164, and miR319 were related to Al-induced PCD [26]. In this study, their target genes are mainly enriched in enzyme activator activity, gluconeogenesis, GTPase activator activity, monosaccharide biosynthetic process, nucleoside-triphosphatase regulator activity, carbohydrate biosynthetic process, etc.

### 2.5. Regulatory Analysis of miRNAs Related to PCD and Target Genes

**Figure 6:**
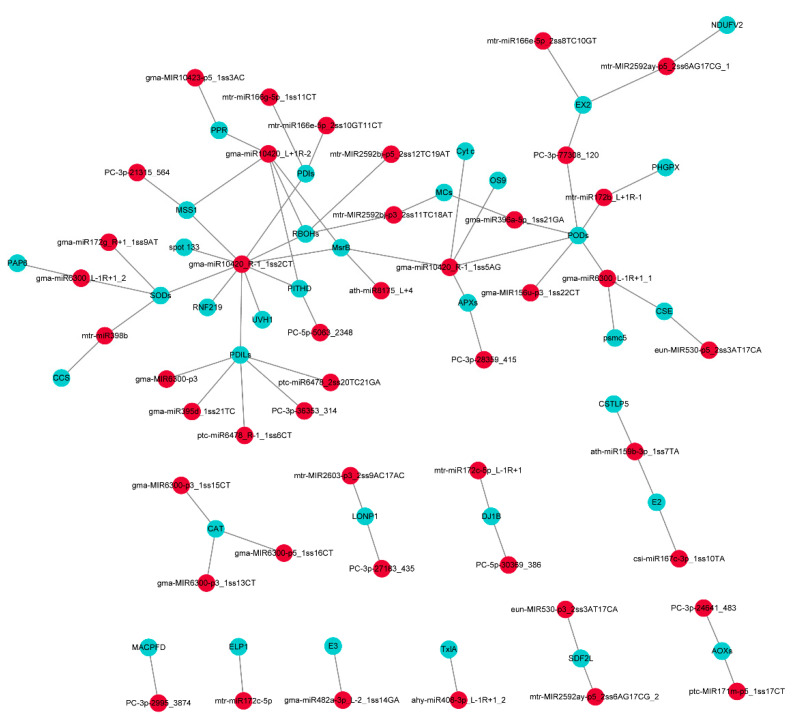
The miRNA-mediated regulatory networks responses to Al stress in peanut. Red circles represent the differentially expressed miRNAs, and blue circles represent the target genes of differentially expressed miRNAs. Abbreviations for target genes are listed in Table S7.

To further investigate the association of Al-induced PCD responsive miRNAs with their targets, we used the Cytoscape platform to analyze the differential miRNAs and their target genes that may be related to PCD, including endoplasmic reticulum stress, oxidative stress, and mitochondrial pathway (Table S7, Figure 6). Eighty-nine pairs of miRNA-mRNA were identified including 69 target genes and 45 miRNAs. The target genes included metacaspases, cytochrome c, cysteine protease, etc. The results indicated that many miRNAs targeted PODs and PDILs, which were involved in oxidative stress and might participate in Al-induced PCD by regulating ROS. Among them, miR10420 and miR6300 jointly regulated PODs. The miR10420 and miR6300 targeted a variety of mRNAs that may be involved in Al-induced PCD, including SODs and PODs, etc. In addition, miR2592 and miR396 jointly regulated the metacaspases (MCs), which are crucial executioners in Al-induced PCD (Table S7, Figure 6). 

### 2.6. Validation of miRNA and Its Target Gene by RT-qPCR 

**Figure 7:**
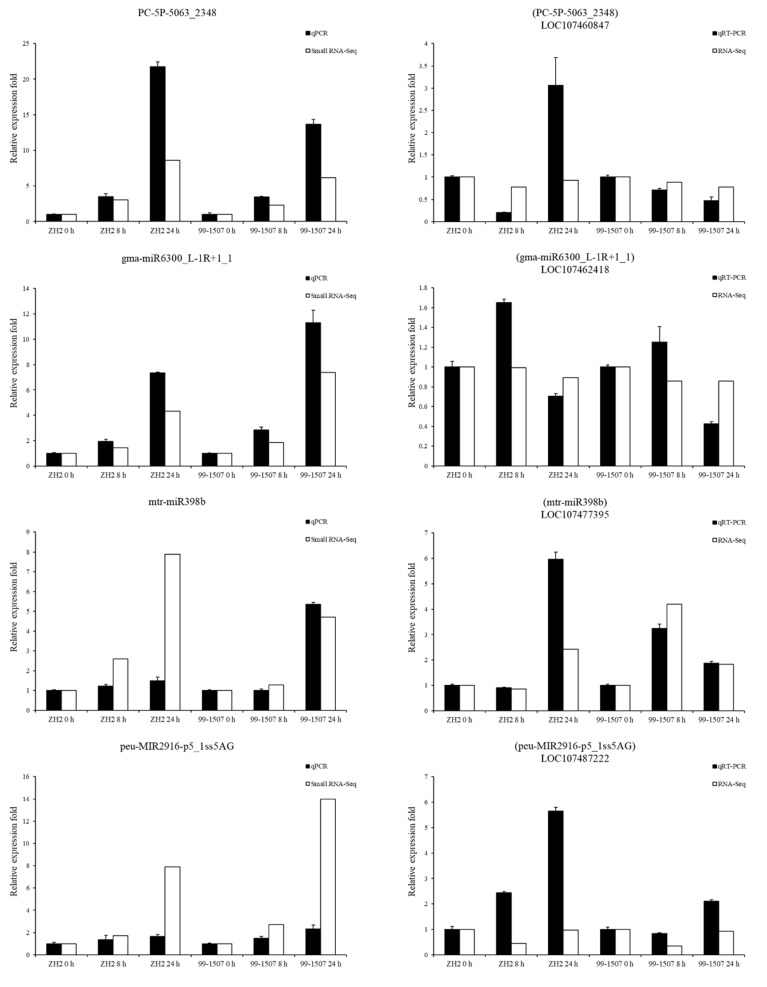

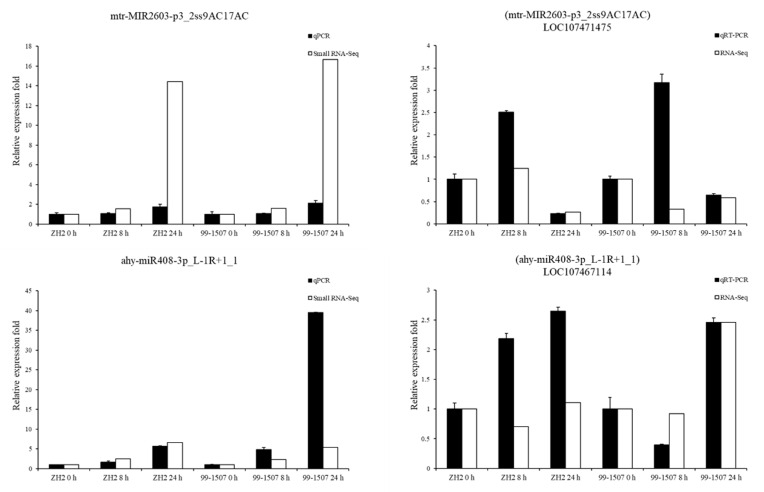
Relative expression results of miRNAs and their targets via qRT-PCR. U6 and actin were used as the internal references for miRNAs and mRNAs, respectively. The normalized miRNA levels and the FPKM values represent the expression level in RNA-seq of each sample, respectively. The expression level at 0 h in each sample was used as a reference state, which was set to 1, and fold change values were shown here.

To confirm the reliability of RNA-Seq data, a validation experiment on randomly selected genes was determined using qRT-PCR (Figure 7). Expression patterns of six miRNAs and six mRNAs under Al stress were consistent with those obtained by the deep-sequencing, which confirmed that our high-throughput sequencing data and subsequent interpretations were reliable.

**Figure 8:**
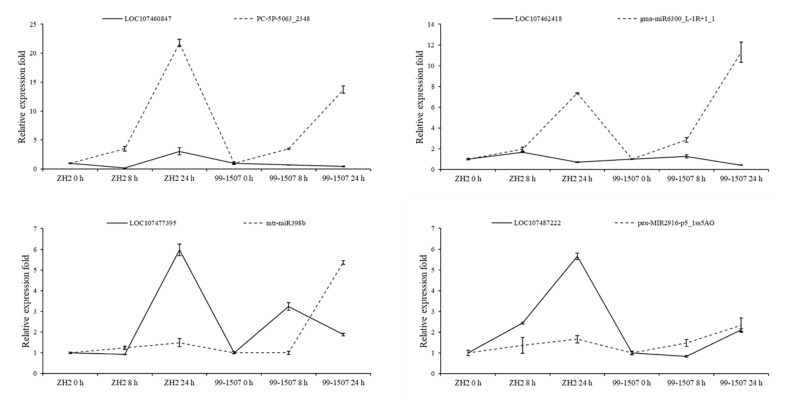

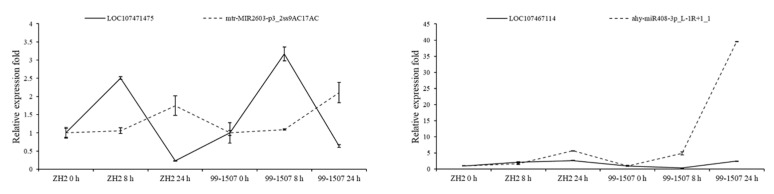
Expression correlation between miRNAs and their targets at six different durations of Al treatment. The thin and thick lines indicate miRNAs and accordingly targets abundance, respectively. To standardize RNA quantity for evaluating relative expression levels, U6 and actin were used as the internal reference genes of miRNAs and their targets, respectively. The expression level at 0 h in each sample was used as reference state, which was set to 1, and fold change values were shown here.

Furthermore, we verified the expression patterns of six miRNA-targets. Stress-responsive miRNA and their targets could sometimes exhibit antagonistic patterns depending on the genotype and stress type (Figure 8). For example, mtr-MIR2603-p3_2ss9AC17AC was downregulated at 8 h and upregulated at 24 h in both ZH2 and 99-1507, and its target LOC107471475 was upregulated at 8 h and downregulated at 24 h in both ZH2 and 99-1507. At some time points, some miRNA-target pairs were presenting a positive relationship at the expression level. It might depend on different varieties and treatment durations.

## 3. Discussion

Recently, some miRNAs from peanut were identified by computational and direct cloning approaches [27]. Ma et al. identified 1082 miRNAs in developing peanut seeds by high-throughput sequencing [28], but the characteristics and functions of most peanut miRNAs are still unknown. Here, we identified 2284 miRNAs in peanut root tips (Table S2). The sRNA length distribution patterns peaked at 24 nt in our study (Figure 1 and Figure S2), which was consistent with previous results for most angiosperms [29]. We found that the sRNA length distribution pattern in peanut was similar to the other plants.

In this study, we identified 147 miRNAs differentially regulated by Al exposure (Table S3). Most of the miRNA members of the same family showed similar expression profiles, for instance, six miR160 and eight miR408 family members were significantly upregulated by Al exposure (Figure 4a,b, Table S3). It has been reported that miR160 could also be upregulated by Al stress in rice [30]. On the contrary, miR160 has been found to be downregulated in barrel medic in response to Al stress [14]. In addition, the miR408 was downregulated by Al exposure in rice [11]. The differential expression patterns of miR160 and miR408 may depend on the different Al treatment conditions and plant species. We also discovered that some previously known miRNAs were regulated by Al stress. Fifty-nine miR6300, 10 miR2592, and six miR530 family members were found to be significantly upregulated by Al stress (Figure 4a,b, Table S3). In sugarcane, miR2592 was likely to be the regulator of internode elongation genes [31]. In plants, miR530 was found to be responsive to abiotic stresses, such as salinity [31]. Our findings implied that the known miRNAs were most likely involved in cross adaptation of plants to abiotic stresses. In our results, miR6300 targeted antioxidant enzymes, including SODs, PODs, and CATs. In addition, miR2592 targeted MCs, which might activate caspases and induce PCD. Moreover, miR530 targeted CSE, which might affect oxidative stress to participate in Al-induced PCD. Their exact functions need to be verified in the future studies. In the meanwhile, different miRNA members within the same family were present at different expression levels after Al exposure. For example, mtr-miR390-R+1 was downregulated by Al exposure, while mtr-miR390_1ss21CA was significantly upregulated (Figure 4a,b, Table S3). Similar events also occurred with miRNA family members in other plants. In radish, miR172a was significantly upregulated, while miR172c was downregulated by Cd exposure [32]. Therefore, a complex mechanism may regulate the expression profiles of members of the same family.

Recent studies have shown that many miRNAs regulate the expression of target genes through transcript cleavage or translation inhibition [33]. A transcriptome-wide analysis of the degradome was performed, and numerous target transcripts for the known and novel miRNAs were determined. This study presented the first transcriptome-based analysis of miRNA targets responsive to Al stress in peanut by degradome sequencing analysis. For the miRNAs, 19237 targets were identified by degradome sequencing (Table S5). An integrated analysis of miRNA expression profiles and their targets could help in identifying the functional miRNA-target modules involved in regulating specific biological processes, such as Al stress and PCD [34]. Consistent with some previous studies, several identified miRNA targets in peanut, belong to a variety of Al-responsive genes, including WRKY factors, ABC transporter, NAC-domain, and F-box proteins (Table S6) [35,36]. It has been previously shown that miR394 targets mRNA of the F-box gene [37]. In this study, ahy-miR394 targeted WRKY transcription factors, ABC transporter, and F-box proteins. WRKY47 has been demonstrated to be required for Al tolerance and root growth via the regulation of cell wall modification genes [38]. AtSTAR1 was required for Al tolerance in Arabidopsis, probably functioning as a bacterial-type ABC transporter by forming a complex with ALS3 [39]. Fang et al. found the degradation of STOP1 mediated by the F-box proteins RAH1 and RAE1 balanced Al tolerance and plant growth [40]. In addition, ahy-miR3508 targeted ABC transporter and NAC-domain in this study. Moreover, it was reported that the positive effect of Al on the growth of rice is mediated by NACs that respond to hormones [41]. The miRNAs involved in the control of gene expression related to PCD were analyzed in our study. Metacaspases (MCs) are cysteine-dependent proteases, which play essential roles in PCD [42]. The mtr-MIR2592bj-p3_2ss11TC18AT and gma-miR396a-5p_1ss21GAg targeted metacaspases, and several miRNAs including miR10420 and miR2592 targeted RBOHs (Table S7). Overexpression of alternative oxidases (AOXs) alleviated mitochondria-dependent PCD induced by Al phytotoxicity in Arabidopsis [43]. In our results, ptc-MIR171m-p5_1ss17CT and PC-3p-24641_483 also targeted AOXs (Table S7).

Recently, several reports have provided evidence that endoplasmic reticulum stress [44], oxidative stress [45], and mitochondrial pathway [46] are associated with PCD. The presence of many signal pathways shows that plants respond to Al exposure through complex mechanisms. By screening the functional annotations of target genes for differential miRNA, the target genes whose functional annotation related to PCD and their corresponding miRNAs were identified. A total of 89 miRNA-mRNA pairs were found (Table S7). The results showed that miRNA targeted genes related to endoplasmic reticulum stress, oxidative stress, and mitochondrial pathway. Other target genes were involved in Al-induced PCD through ubiquitination. It has been shown that the ubiquitin-26s proteasome system (UPS) is an important protein degradation system in cells. Under the joint of ubiquitin-activating enzyme (E1), ubiquitin-conjugating enzyme (E2), and ubiquitin ligase (E3), the system binds the tag protein ubiquitin composed of 76 amino acids to the substrate protein. The labeled protein is degraded by proteasome or regulated by signal pathway. Different forms of ubiquitination modification of substrate proteins can regulate a variety of cell activities, such as signal transduction, gene transcription, and PCD [47]. Several studies showed that UPS plays a key and complex role in the regulation of PCD [48]. Among them, the RING finger E3 family (RING finger) has the closest relationship with PCD [49]. In our results, ath-miR159b-3p_1ss7TA and csi-miR167c-3p_1ss10TA might target the ubiquitin-conjugating enzyme E2 in response to Al-induced PCD in peanut. The miR159 could respond to Al stress [50] and inhibited GAMYBs transcription factor by inhibiting GA-regulated PCD in tapetum cells [51]. The miR167 negatively regulated ARF, which encoded auxin response transcription factors [52]. In *B. napus*, BnNRAMP1b was a target of miR167 under Cd stress [53]. The miR482 may be involved in Al-induced PCD by targeting E3 ubiquitin-protein ligase SINAT5 (Table S7), it was found to be involved in the defense regulation of plant pathogens [54]. The miR10420 may be involved in Al-induced PCD by targeting 26S protease regulatory subunit and RING finger protein (Table S7). There are few studies on miR10420.

From our findings, miRNA may be involved in Al stress by targeting ubiquitination-related genes and then activating caspase in coordination with the mitochondrial pathway to induce PCD. However, some specific mechanisms are still controversial, which will be a hot issue in the future. In addition, most of the previous studies of miRNA in Al stress focused on humans and other animals [55], but few are available on plants. The inhibition of proteasome function by gene silencing could also cause PCD in tobacco cells [56]. The ubiquitin-26s proteasome system of ubR48 (ubiquitin variant, the 48th lysine of the translated peptide chain is replaced by arginine) in transgenic *Arabidopsis thaliana* was inhibited, which induced PCD [57]. There is an important role of UPS in PCD regulation. However, the regulatory process and its components in the control of PCD in plants should be studied in the future.

## 4. Materials and Methods

### 4.1. Plant Materials and Al Stress Treatment

Two different peanut (*Arachis hypogaea L.*) varieties 99-1507 (T, Al-tolerant) and ZH2 (S, Al-sensitive) were selected as plant materials. The seeds were first incubated in the moist perlite under dark conditions at 26 ± 1 °C for 3–4 days for germination. After germination, the seed coats were removed, then seedlings with about 2–3 cm roots length were transferred in a modified Hoagland nutrient solution. The growing conditions were set as 26 ± 2 °C, 12 h light/12 h dark and the light intensity was 30–50 μM·m^−2^·s ^−1^. Seedlings with the third true leaf were pretreated with 0.1 mM CaCl_2_ solution (pH 4.2) for 24 h, then treated with 100 μM AlCl_3_ solution (containing 0.1 mM CaCl_2_, pH 4.2) for 0, 8, and 24 h. Thereafter, they were treated with 0.1 mM CaCl_2_ for the same time (8 and 24 h-0) as the mock. Approximately 1 cm of the root tips were gained for the sample collection. Each treatment was carried out in triple biological replicates except the control 8 and 24 h-0, which were repeated twice. All of the samples were immediately frozen in liquid nitrogen and stored at −80 °C.

### 4.2. The sRNA Sequencing and miRNAs Identification

Twenty six small RNA libraries were constructed using the TruSeq Small RNA Sample Prep Kits (Illumina, San Diego, CA, USA), according to the manufacturer’s instructions. Total RNA, which was a qualified sample, was ligated to the RNA 3′ and RNA 5′ adapters and reversed transcription, followed by PCR to make cDNA constructs of the small RNAs. Finally, the cDNA constructs were purified and the library was validated. Then, single-end sequencing (50 bp) was performed on an Illumina Hiseq2500 at the Hangzhou LC-Bio Co., Ltd. (Hangzhou, China). Thereafter, the dataset was processed using an in-house program, ACGT101-miR (LC Sciences, Houston, TX, USA) to remove adapter dimers, low complexity reads, common RNA families (rRNA, tRNA, snRNA, snoRNA), and RepBase database (http://www.girinst.org/repbase, accessed on 27 December 2021, repetitive sequence database). Subsequently, unique sequences with 18~25 nucleotides in length were mapped to specific species precursors in miRBase 22.0 (http://www.mirbase.org/, accessed on 27 December 2021) by BLAST search to identify known miRNAs and novel 3p- and 5p- derived miRNAs.

### 4.3. Analysis of Differentially Expressed miRNAs

To discover the expression profiles of the target genes, 26 independent libraries were constructed from the RNA samples of two varieties from five different Al treatments. For each library, all of the sequences were processed to filter out adaptor and lower quality sequences. Then, all of the valid data were performed for further miRNA identification and prediction analysis. Differential expression of miRNAs based on normalized deep-sequencing counts was analyzed by selectively using the Fisher exact test, Chi-squared 2 × 2 test, Chi-squared n × n test, Student *t*-test or ANOVA based on the experiments design. Then, a rigorous algorithm method was performed to identify the differentially expressed genes between two samples. The significantly differentially expressed genes among all of the different samples were judged by the threshold as follows: *p*-value < 0.05 and the absolute value of log2 ratio ≥ 1. Differentially expressed miRNAs were identified using UpSetR (v1.4.0) (LC-Bio Co., Ltd., Hangzhou, China) in R. Through mock, the influence of unrelated factors was eliminated. The heatmap of the differentially expressed miRNAs in ZH2 and 99-1507 was constructed using the ggplot2 package (v1.0.12) in R.

### 4.4. Degradome Sequencing, Target Identification, and Analysis

An equal number of three biological replicates of RNA samples at the same time point were mixed together to generate six degradation libraries. Then, 26 sRNA samples and six degradation mixed samples were sent to Hangzhou LC-Bio Co., Ltd. (Hangzhou, China). The extracted sequencing reads generated from degradome sequencing were used to identify potential cleavage targets through the CleaveLand (v4.0). Then, the degradome reads were mapped to the peanut transcriptome data, which was previously tested in our laboratory [24].

### 4.5. Gene Annotation, Functional Enrichment, and Pathway Enrichment Analysis

Expression analysis of all the target genes was performed using transcriptomic data of peanut in response to Al stress [24]. GO functional classification of all the identified targets of differentially expressed miRNAs was performed to uncover the miRNA-gene regulatory network. The KEGG pathway for the target genes was analyzed. Target genes were annotated based on the Gene Ontology database (https://ftp.ncbi.nlm.nih.gov/gene/DATA/gene2go.gz, accessed on 27 December 2021). Pathway analyses of target genes were performed using The Kyoto Encyclopedia of Gene and Genome (KEGG) database (http://www.genome.jp/kegg). Differential miRNA-mRNA related to Al-induced PCD was presented by functional annotation and the miRNA-mRNA network construction was presented using Cytoscape (v3.4.0). For network construction, stress-responsive miRNAs and genes, whose functional annotation were involved in PCD response, were included.

### 4.6. Verification by qRT-PCR

For validation of miRNAs and their targets expression, high-throughput sequencing technology and qRT-PCR of randomly selected miRNAs and genes were performed. The qRT-PCR analysis was carried out for six miRNAs and six targets at three time points (0, 8, and 24 h). The qRT-PCR analysis was performed with the miRNA 1st Strand cDNA Synthesis Kit (Vazyme Biotech, Nanjing, China) and SYBR Premix Ex TaqTM (TaKaRa, Dalian, China), according to the manufacturer’s instructions. RT-qPCR was conducted on a Bio-Rad iQ5 Real-time PCR System (Bio-Rad Laboratories, Hercules, CA, USA). U6 was used as the internal control for miRNAs and actin was used as the internal control for target genes to standardize the RNA quantity for evaluating relative expression levels. All of the primers are listed in Table S8. At least three technical replicates were carried out for each sample and three independent biological replicates were performed.

## 5. Conclusions

**Figure 9:**
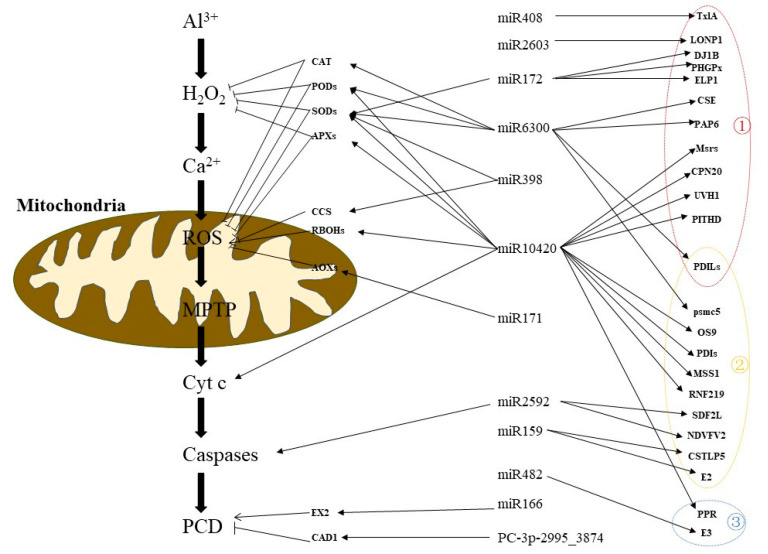
Proposed working model for PCD induced by Al. The sharp head represents promotion, whereas the flat head represents suppression. ROS: Reactive oxygen species; MPTP: Mitochondrial permeability transition pore; Cyt c: Cytochrome C; Caspases: Cysteinyl aspartate specific proteinase; PCD: Programmed cell death; numbers enclosed in circles in the figure represent: ①, oxidative stress; ②, endoplasmic reticulum stress; ③, programmed cell death regulation. For detailed explanation, see Table S7 and Figure 6.

In summary, we conducted an integrated analysis of the sRNAs and degradome to generate a comprehensive resource focused on identifying the key regulatory miRNA-target for Al-induced PCD. A total number of 2284 miRNAs were identified in peanut. In addition, 147 miRNAs were significantly differentially expressed and identified as Al-responsive miRNAs. We screened the target genes related to endoplasmic reticulum stress, PCD, oxidative stress, etc. Eighty nine pairs of miRNA-mRNA composed of 45 differential miRNAs and 69 target genes were identified (Figure 6, Table S7), which may be the key genes related to Al-induced PCD. Based on our small RNA, degradome sequencing, previous transcriptomics analysis, and results of other peanut crops, a putative model of regulatory network associated with Al-induced PCD in peanut was proposed (Figure 9). Al stress triggered H_2_O_2_ bursts, which increased calcium concentrations to the point of overload. This triggered ROS production in mitochondria, opened MPTP, released cytochrome c, activated caspases activity, and finally induced the occurrence of PCD. Many miRNAs are involved in this process via their regulation of mRNAs related to PCD. For example, miR10420 targeted their target genes to participate in oxidative stress, endoplasmic reticulum stress, and Cyt c releasing, etc. Through endoplasmic reticulum stress, and the miR2592 targeted MCs are involved in Al-induced PCD. Herein, our results will help in elucidating the miRNA-mediated molecular mechanisms of Al-induced PCD in peanut and in providing insights for plants adaption to Al stress.

## Supplementary Materials

The following supporting information can be downloaded at: https://www.mdpi.com/article/10.3390/ijms23010246/s1.
