# Supplementary material for: Integration of Small RNA and Degradome Sequencing Reveals the Regulatory Network of Al-Induced Programmed Cell Death in Peanut
Source: Int J Mol Sci. 2021 Dec 27;23(1):246. doi: 10.3390/ijms23010246 (PMC8745729; doi:10.3390/ijms23010246)
Supplement: Supplementary file 1 [file ijms-23-00246-s001.zip › Fig S2 Length distribution of counts of total and unique sRNA in this study.pdf]

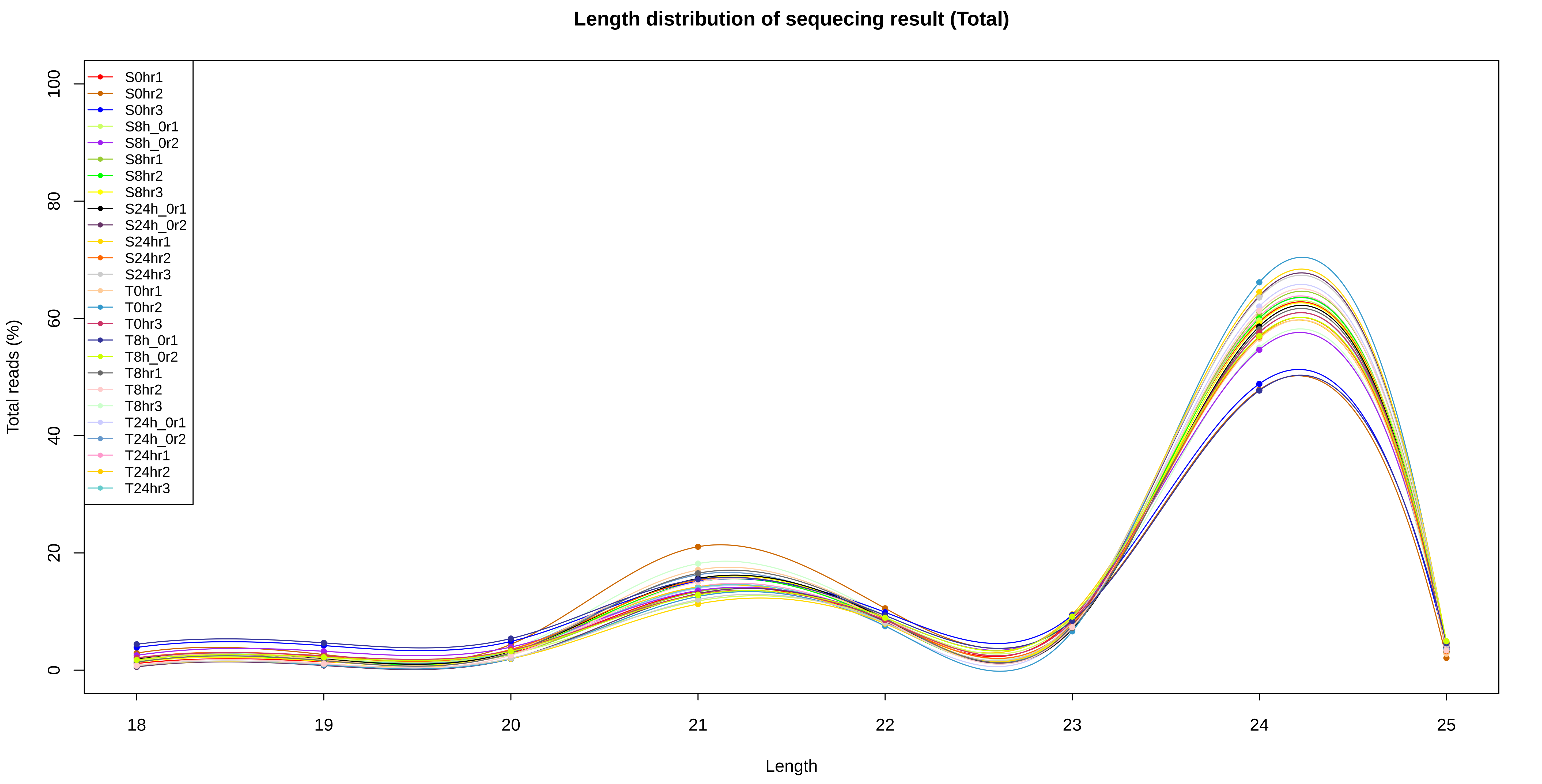

Length distribution of sequecing result (Total)
Total reads (%)
0
20
40
60
80
100
18
19
20
21
22
23
24
25
Length
S0hr1
S0hr2
S0hr3
S8h_0r1
S8h_0r2
S8hr1
S8hr2
S8hr3
S24h_0r1
S24h_0r2
S24hr1
S24hr2
S24hr3
T0hr1
T0hr2
T0hr3
T8h_0r1
T8h_0r2
T8hr1
T8hr2
T8hr3
T24h_0r1
T24h_0r2
T24hr1
T24hr2
T24hr3

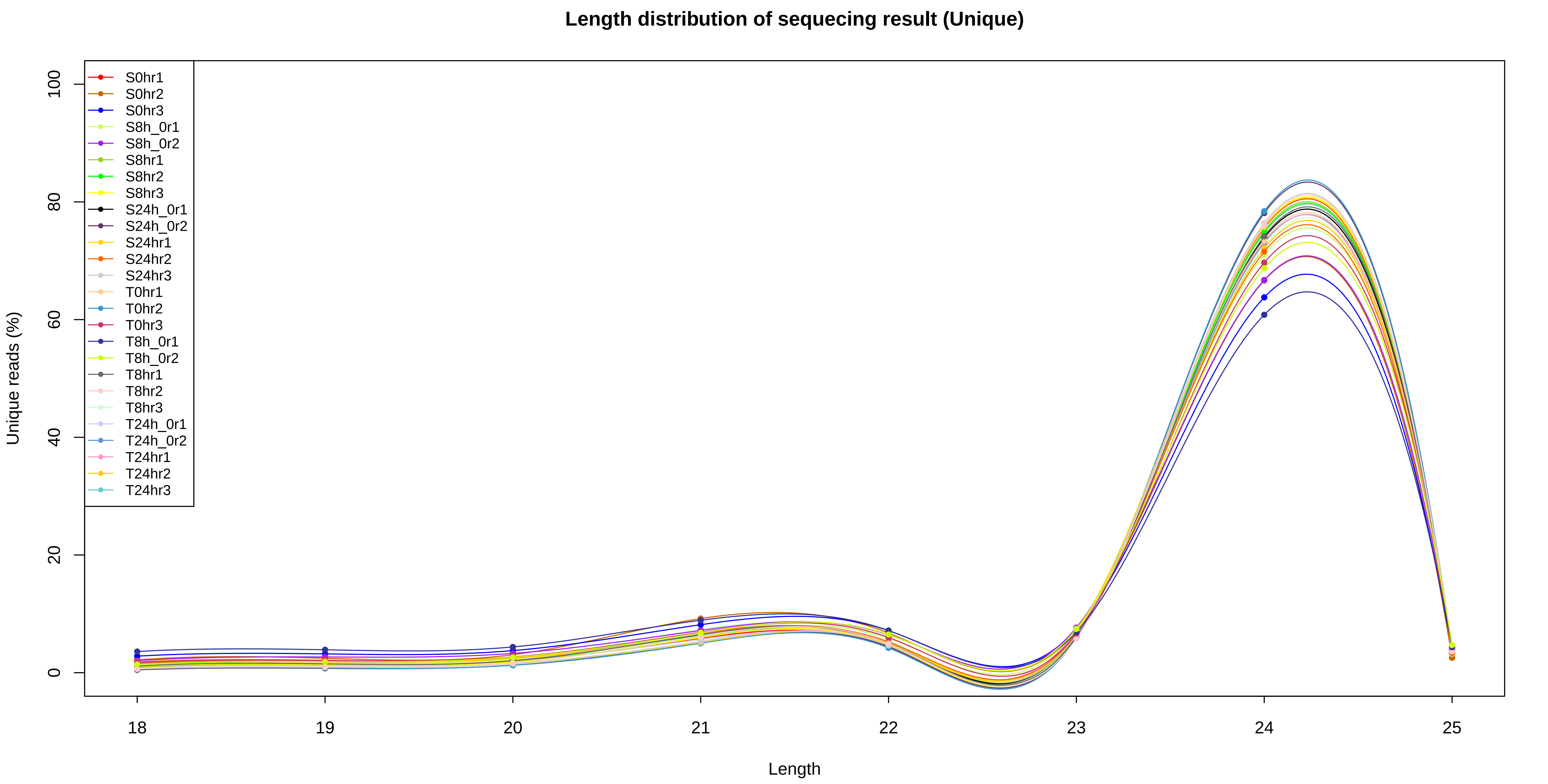
Length distribution of sequecing result (Unique)
Unique reads (%)
Length
0
20
40
60
80
100
18
19
20
21
22
23
24
25
S0hr1
S0hr2
S0hr3
S8h_0r1
S8h_0r2
S8hr1
S8hr2
S8hr3
S24h_0r1
S24h_0r2
S24hr1
S24hr2
S24hr3
T0hr1
T0hr2
T0hr3
T8h_0r1
T8h_0r2
T8hr1
T8hr2
T8hr3
T24h_0r1
T24h_0r2
T24hr1
T24hr2
T24hr3
