# Supplementary figures and images for: Integration of Small RNA and Degradome Sequencing Reveals the Regulatory Network of Al-Induced Programmed Cell Death in Peanut
Source: Int J Mol Sci. 2021 Dec 27;23(1):246. doi: 10.3390/ijms23010246 (PMC8745729; doi:10.3390/ijms23010246)

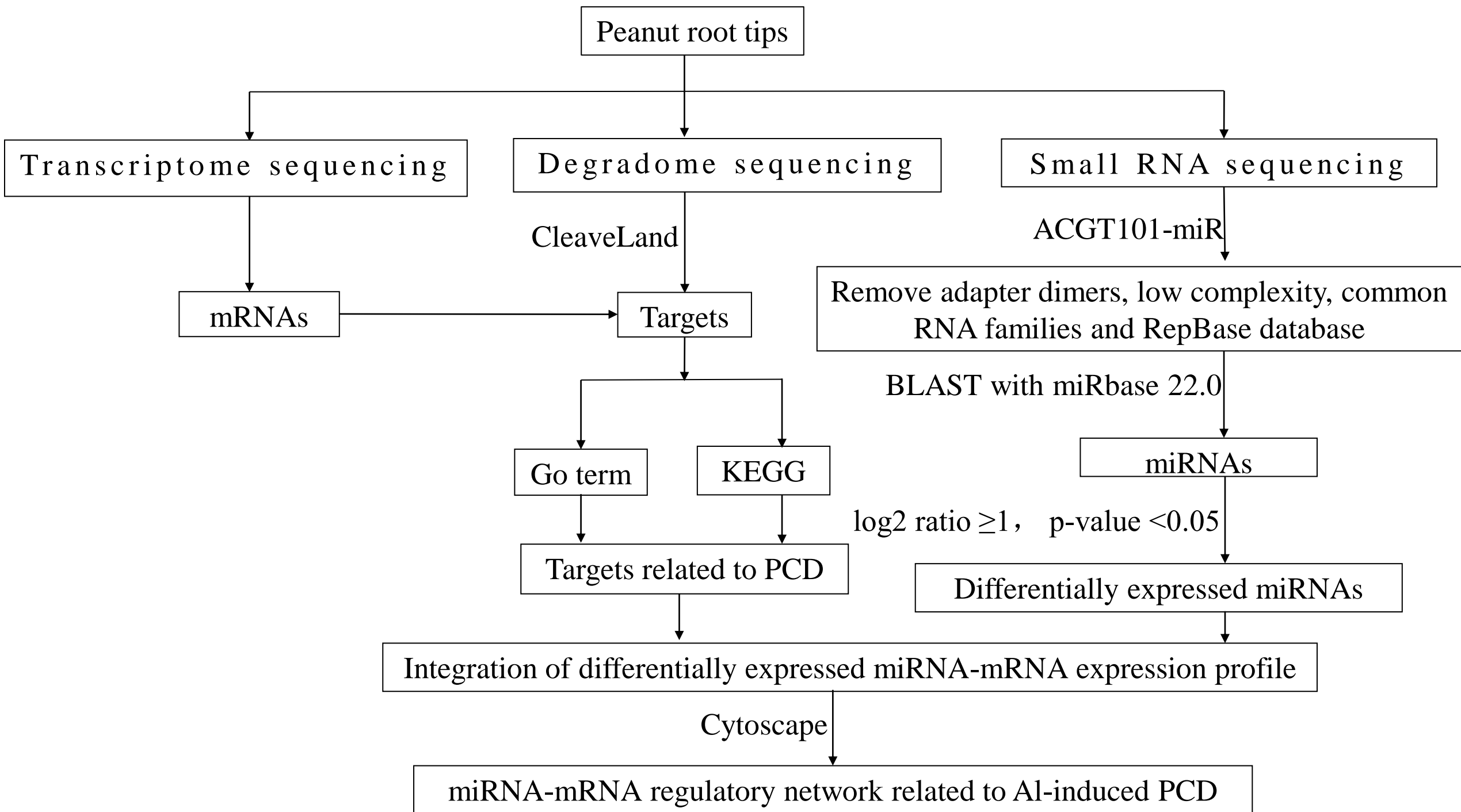

Supplement: Supplementary file 1 [file ijms-23-00246-s001.zip › Fig S1 The developmental bioinformatics pipeline of data sequencing analysis in this study.pdf]

Number of miRNA

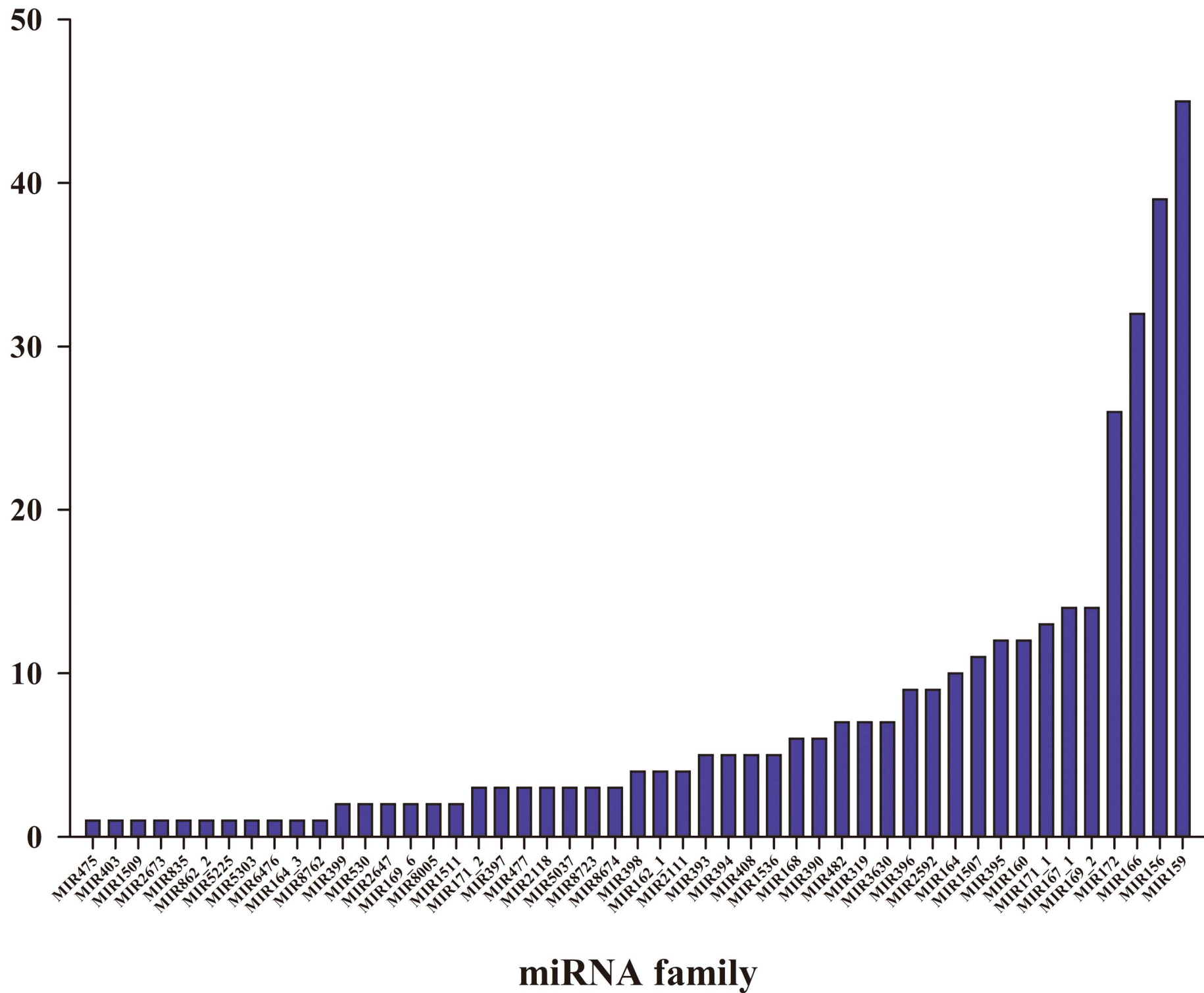

Supplement: Supplementary file 1 [file ijms-23-00246-s001.zip › Fig S3 The number of miRNA family members.pdf]
